# Frequent Self-Weighing and Visual Feedback for Weight Loss in Overweight Adults

**DOI:** 10.1155/2015/763680

**Published:** 2015-05-12

**Authors:** Carly R. Pacanowski, David A. Levitsky

**Affiliations:** ^1^University of Minnesota Division of Epidemiology & Community Health, 1300 S. 2nd Street, Suite 300, Minneapolis, MN 55454, USA; ^2^Cornell University Division of Nutritional Sciences, 112 Savage Hall, Ithaca, NY 14853, USA

## Abstract

Evidence has suggested that self-weighing may be beneficial for weight control in adults, but few studies have independently assessed the contribution of this behavior to weight loss. This study experimentally tested daily self-weighing and visual feedback (the Caloric Titration Method (CTM)) as a weight loss and weight loss maintenance intervention over 2 years. 162 overweight individuals were randomized to the CTM intervention or delayed treatment control group. In year 1, weight change was compared between groups, and in year 2, the control group started using the CTM while the intervention group continued using the CTM for maintenance. A significant difference in weight loss over the first year (CTM *n* = 70; 2.6 ± 5.9 kg versus control *n* = 65; 0.5 ± 4.4 kg, *p* = 0.019) was qualified by a group × gender × time interaction (*p* = 0.002) such that men lost more weight using the CTM. In year 2, the CTM group maintained their weight and the control group lost an amount similar to the intervention group in year 1. Daily self-weighing and visual feedback facilitated a minimal amount of weight loss and maintenance of this loss. Future research investigating characteristics of those who benefit from this type of self-directed intervention is warranted.

## 1. Introduction

Several studies have included self-weighing as a component of behavioral weight loss interventions [1–10]. Because self-weighing is typically used with other techniques to promote weight loss, its effectiveness has not been thoroughly assessed independently. Early studies compared groups that differed only in self-weighing frequency and indicated that the behavior was not helpful for weight loss [3–7]. However, more recent evidence has suggested that frequent self-weighing may be beneficial for weight control, including weight loss, prevention of weight gain, and prevention of weight regain after loss ([1, 2, 8, 11–16]; reviews: [17, 18]). The majority of this evidence, with the exception of Fujimoto et al. (1992) and Steinberg et al. (2013), is correlational making it inappropriate to infer causality regarding the role of self-weighing in weight control.

Isolating the effects of frequent self-weighing for weight loss in adults is important for the field of weight control. Despite evidence that increases in overweight and obesity may be decreasing in some categories of the population, the proportion of overweight and obese adults in the United States remains high [19]. A weight loss as small as 5% of body weight may improve health [20]. Because frequent self-weighing is both relatively affordable and not time consuming, it is an important method to test independently to produce sustained weight reduction. Moreover, it is a feasible technique for individual use to monitor progress, with or without the assistance of a healthcare professional.

This study tests the effectiveness of a simple and affordable behavioral technique, frequent self-weighing and visual feedback, termed the “Caloric Titration Method” or CTM for weight loss and maintenance of loss in overweight and obese adults over a two-year period. It was hypothesized that the intervention group, given tools to self-weigh and view visual feedback of their weight history, would lose more weight over a one-year period compared with a delayed control group. Additionally, it was hypothesized that the intervention group would maintain their weight loss during the second year of the trial due to continued weighing and weight feedback.

## 2. Methods

### 2.1. Participants and Procedures

All study procedures were conducted after approval from the university institutional review board. One hundred and seventy-eight individuals responded to advertisements, soliciting adults interested in weight loss. Newspaper advertisements, email newsletters, and public service announcements described study eligibility criteria (i.e., potential participants should be ≥18, be not pregnant or planning to become pregnant, not have diabetes, not have a current eating disorder or history of an eating disorder, and have a body mass index (BMI) ≥27.0 kg/m^2^). Sixteen interested individuals did not meet the BMI cutoff and were invited to participate in a “weight maintenance” cohort; results are discussed elsewhere [21].

Interested individuals were sent a copy of the consent form before attending the initial meeting. They were randomly assigned to one of two groups (control or intervention) and offered to attend one of two sessions for either group, held on different days (to maximize attendance). Participants were not informed of their group assignment until the initial session to minimize control group dropout and were asked to voluntarily consent after the session.

The initial session was an educational presentation about evidence-based strategies for weight loss, with an emphasis on self-selection of strategies to meet individual needs. Making small changes, amounting to or averaging 100 kcal deficits per day, was encouraged (e.g., skipping dessert a few times per week; using a meal replacement for lunch 3x a week; abstaining from snacking most days of the week). For more details on evidence-based strategies that were presented as options, see reference [22]. The only difference between control and intervention group initial sessions was that the intervention group session concluded with an explanation of the CTM intervention. Participants were provided with a typical bathroom scale (American Weigh Scales Model 330 LPW) and asked to weigh daily, under consistent circumstances, ideally, first thing in the morning, immediately after rising. They were also shown how to access a computer website (http://weightloss.human.cornell.edu/) where they were directed to register and enter their weight daily.

Control group participants were told that they would receive the intervention after one year. During this year, they were permitted to do anything they would normally do to lose weight. Intervention participants were permitted to do anything they wished to lose weight in addition to using the CTM. A goal of 10% weight loss in the first year was advised and then maintenance through continued weighing and recording in the second year.

After one year, participants randomized to the control group were provided with the CTM intervention—a body weight scale, instructions for setting up an account using the website, and the same informational handout. The audio-recorded educational session and explanation of how to use the CTM were available on the website. Participants in the intervention group were instructed to continue weighing themselves and entering their weight during year 2 of the trial and to maintain their weight loss or continue losing weight if they wished.

### 2.2. The Caloric Titration Method (CTM) Intervention

**Figure 1:**
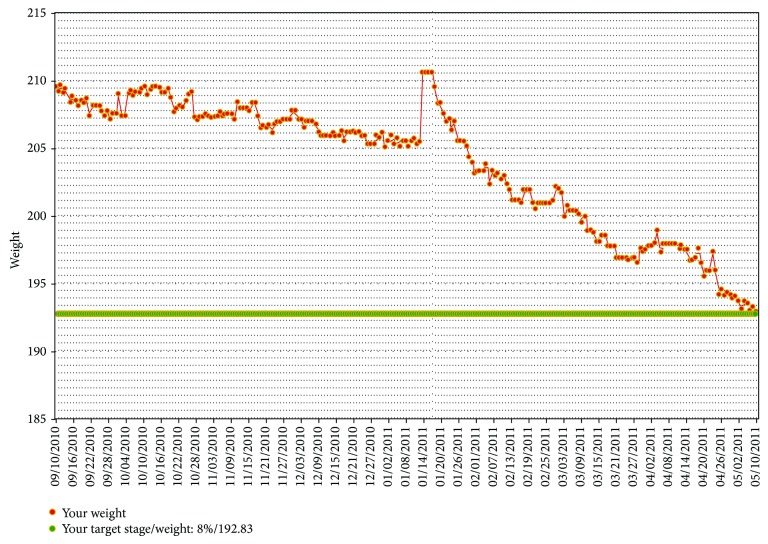
Sample view of CTM weight graph.

The CTM provides feedback of an individual's weight trends over time, as shown in Figure 1.

Weight loss is directed by small decrements, equivalent to 1% of starting body weight. Once 8 weight measurements have been entered, a green line appears 1% below the user's current weight to show the target weight. After the user reaches and maintains the target weight for 8 days, the green line is reduced by another 1% on the chart. This procedure continued until a maximum of 10% loss is reached, at which point weight maintenance is recommended. Intervention participants were directed to aim for the 10% weight loss goal over the first year, at which time they would maintain this loss during the second year of the trial. If participants did not enter a minimum of 3 weights per week, they were sent an email reminding them that they had not entered a sufficient number of weights for that week.

The CTM prompts slow weight loss in hope of producing a sustained weight loss. This method of small changes [23] and slow weight loss is contradictory to the idea that producing a rapid initial weight loss is more effective in producing and sustaining a weight loss [24, 25]. The CTM allows people to test making small decrements in their energy intake such as a reduction in portion size, snacking, or desert eating by viewing feedback. By viewing the daily graph of their weight, they will know if the change they made is sufficient to produce a small decrement in their weight. Through this process of trial and error, each participant can discover their own methods for producing and sustaining a small energy deficit.

### 2.3. Measures

#### 2.3.1. Body Weight

Participants were weighed using the same LifeSource Precision Scale, Model UC-321 at the initial session (baseline) and at 6, 12, and 24 months after baseline. Weight measurements were conducted in campus buildings or in a public location of the participant's choice.

#### 2.3.2. Height

Height was self-reported at the initial session and at the 6-month weigh-in. If participants reported disparate heights, an average was taken. In addition, if participants visited a doctors' office and had their height measured, they were encouraged to contact the researchers with this information.

#### 2.3.3. Demographic and Psychological Characteristics

Online questionnaires were administered at each of the time points listed above. The questionnaires assessed psychological and behavioral factors and experience using the CTM and are discussed elsewhere [26].

#### 2.3.4. Analysis

Descriptive statistics and independent samples *t*-tests were performed; mixed models were used to analyze the data more extensively. All analyses followed an intent to treat strategy.

Some participants only have weight measurement values at baseline and 6 months due to nonresponse after repeated contacts from the research team. Because these individuals confirmed participation and were randomized, any information (e.g., survey or first weight) was included in analyses when possible. For example, in cases where the individual only attended the initial session, their initial session weight was carried forward, giving them a weight change over the first year of “0,” a method known as last observation carried forward (LOCF). Analyses were repeated using a variable that excluded individuals with missing 12-month weights and a variable that carried their baseline or 6-month weight forward.

All *t*-tests were 2-tailed because it was reasoned a priori that the results could go in either direction (the control group could lose more weight than the CTM intervention group or the CTM intervention group could lose more weight than the control group).

Mixed models allow for maximal usage of missing data; if an individual had only 2 data points for the first year, their data could be used without imputation. Specifically, a random intercept random slope model was employed, which computes a linear regression for each individual's weight trajectory over the specified time period, allowing for more accurate description of their weight trajectory. The mixed model included a main effect of treatment group, a main effect of time, and the interaction between time and treatment group. The interaction term answers the question “Did weight change differently according to group?” Time was analyzed as a continuous variable.

## 3. Results

Of the 162 participants who were randomized (88 to the CTM intervention group and 74 to the control group, resp.), 8 never attended an initial session and 4 contacted the researchers after being randomized to say that they did not meet inclusion criteria (e.g., had diabetes and did not notice that was an exclusion criterion). Chi-squared statistics did not reveal significant differences as to which group the no-shows were randomized.

### 3.1. Participant Characteristics

Table 1 displays baseline participant characteristics.

The sample had an average age of 46.6 ± 9.8 years and an average BMI of 33.5 ± 5.1 kg/m^2^ and had completed an average of 15.9 ± 2.2 years of education (range 12–19 years). The majority of participants (81.9%) were female. Most participants self-identified as white; the racial/ethnic composition of the sample is presented in Table 1. There were no significant differences between the control and intervention group at baseline.

### 3.2. Year 1 Results

On average, more than 4 weights per week were entered into the CTM program. A significant difference in mean within subject weight loss was found between the control group (*n* = 65; 0.5 ± 4.4 kilograms (kg)) and the CTM intervention group (*n* = 70; 2.6 ± 5.9 kg) over the first year (*p* = 0.019). Nonparametric tests (Mann-Whitney *U*) revealed very similar results (*p* = 0.02).

The difference in weight loss remained when employing the LOCF method for handling missing data. A significant difference in mean within subject weight loss was found between the control group (*n* = 67; 0.4 ± 4.4 kg) and the CTM intervention group (*n* = 81; 2.1 ± 5.6 kg) over the first year (*p* = 0.037).

The number of participants achieving ≥ 5% weight loss over the first year in the intervention group was 20 (28.6%) and 7 (10.8%) in the control group (chi-squared 2-sided, *p* value = 0.01). The number of participants achieving ≥10% weight loss over the first year in the intervention group was 6 (8.6%) and 3 (4.6%) in the control group (Fisher's exact test 2-sided, *p* value = 0.50).

A mixed model analyzing weight change over the first year revealed a significant interaction between treatment group and time (*p* = 0.026). When comparing the linear weight trajectories between the intervention group and the control group, the slope of the line increases by 1.0 kg (95% CI [0.1–1.9]).

**Figure 2:**
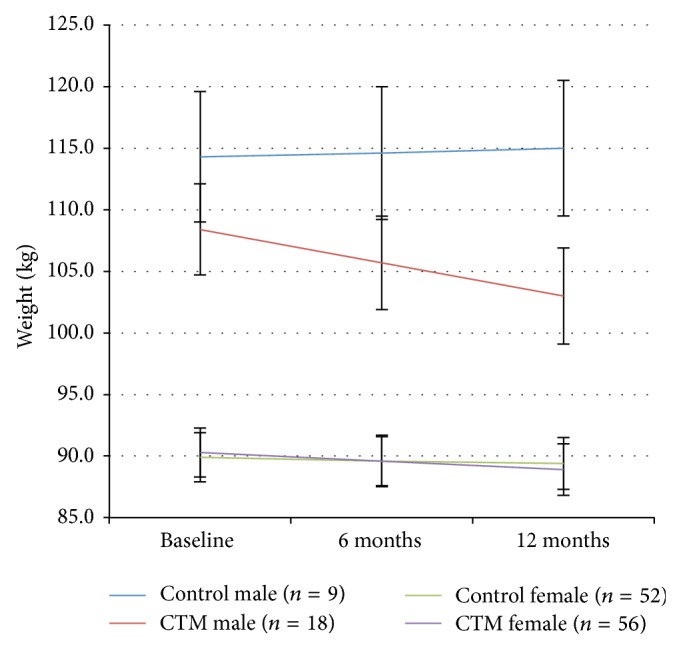
Body weight over time by treatment group and gender: year 1. Error bars are ±1 standard deviation of the estimated marginal mean for the mixed model.

Exploratory analyses revealed that gender was influencing the difference in weight loss over the first year.  Figure 2 graphs the mixed model's estimation of means by treatment group and gender at each time point and displays the significant three-way interaction between treatment group, gender, and time (*p* = 0.02).

The difference in baseline body weight in men randomized to the CTM intervention and control group was not significantly different (*p* = 0.314). Due to the possibility that outliers were driving the group by gender interaction, residual versus predicted values of the dependent variable, weight change over the first year, were plotted and appeared fairly evenly dispersed around the horizontal axis.

### 3.3. Year 2 Results

In year 2, participants in the control group were provided with the CTM intervention. This group's weight loss over year 2 was on average 1.9 ± 5.7 kg (*n* = 57). This loss was significantly different from zero (*p* = 0.013) but was not significantly different from the average loss of the CTM intervention group in year 1 (2.6 ± 5.9 kg; *n* = 70; *p* = 0.524).

In year 2, the goal for participants continuing in the CTM intervention group was maintenance. The average weight change was 0.1 ± 4.8 kg, a value not significantly different from zero (*p* = 0.929).

**Figure 3:**
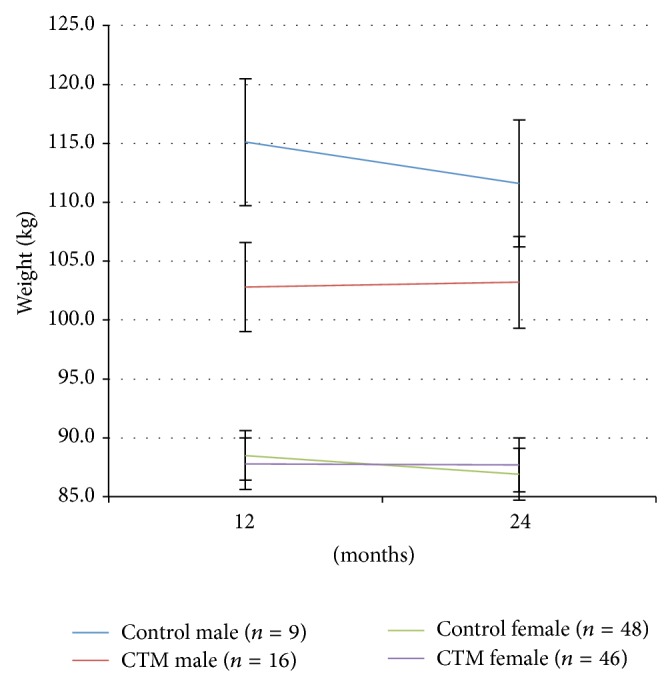
Body weight over time by treatment group and gender: year 2. Error bars are ±1 standard deviation of the estimated marginal mean for the mixed model.

Figure 3 displays change in weight over time by treatment group and gender using means from a random intercept model of weight trajectory between 12 and 24 months.

The males continuing to use the CTM intervention maintained their reduced weight, while the control males (given the intervention) lost weight—this loss was not statistically different from the amount lost by the males given the CTM intervention in the first year (*p* = 0.42). The females in the second year, on the other hand, showed no significant effect of using the CTM similar to the effect observed during the first year.

**Figure 4:**
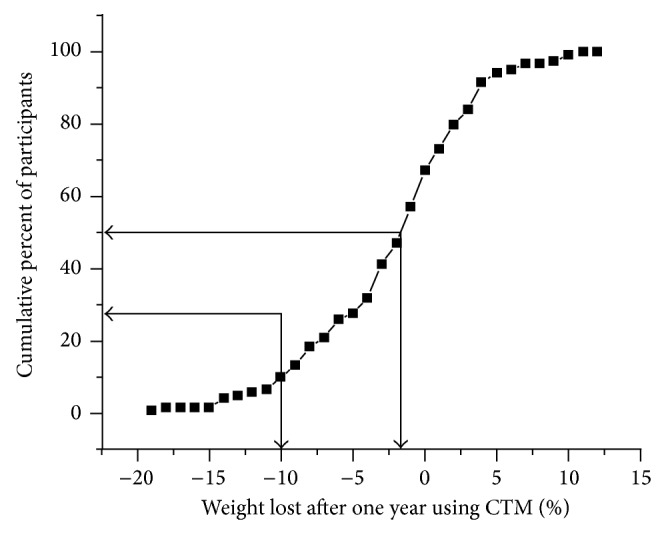
Cumulative distribution of weight loss after one year of CTM use.

Though the goal of the CTM intervention group was to lose 10% of starting weight over one year, most of the participants did not reach this goal.  Figure 4 shows the cumulative distribution of weight loss for participants using the CTM over one year, combining the first year of the intervention group with the second year of the delayed control group for a sample size of 119.

The average weight loss was 2.5 ± 5.7 percent of starting body weight.

## 4. Discussion

The major finding of this study is that the use of frequent weighing accompanied by visual feedback of weight, without a prescribed diet or exercise plan, was effective in producing a small but sustainable weight loss in overweight males.

The amount of weight lost during the first year of the intervention was relatively small; intervention participants lost an average of 2.7 ± 5.9 percent of their body weight, while participants in the control group lost an average of 0.5 ± 4.8 percent of their body weight. A similar degree of weight loss was observed in the delayed treatment control group, who lost an average of 1.9 ± 5.4 percent of their starting weight over year 2 of the trial. For comparison with published results, over the first 6 months, intervention participants lost an average of 1.1 ± 4.2 percent of their body weight and control participants lost an average of 0.1 ± 3.6 percent of their body weight. Two studies reported, on average, greater than 6 percent of body weight lost after 6 months, using an intervention including self-weighing but also other education and behavioral components [2, 8]. The amount of weight loss observed in this study over one year (2.7 ± 5.9 percent) was comparable to programs using internet based weight loss with minimal intensity (e.g., 1.1 percent weight loss at 1 year using a commercial online program in “Internet-Based Commercial Weight Loss Programs” section [27]).

For the first year of the study, exploratory analyses led to post hoc findings suggesting that the CTM helped to facilitate weight loss in men, but to a lesser degree, and not statistically significant in women. Counter to expectations, women in the control group lost weight during the trial, albeit a small amount. It is possible that the sample size was too small to detect a significant between-group effect or that this method was simply not effective beyond what women normally would do to lose weight. For males, the contrast is clearer—males in the control group gained weight over the first year while males in the experimental group lost weight.

Year 2 of the study provided data to support the hypothesis that after facilitating weight loss, the CTM would help individuals to maintain that loss.  Table 2 shows the mean percent weight gain from the present study along with data from 44 groups collected from 12 published studies that tracked body weights for one year following weight loss. As is evident from this table, the maintenance of body weight following weight loss is rarely observed. The average amount of weight regain was 35.5% (95% CI [20.91–50.16]) of the participant's weight measured at the end of the weight loss treatment. These data suggest that the use of the CTM, or other methods that utilize daily weighing, may play a greater role in the prevention of weight regain than in the production of a weight loss.

Principles of behaviorism underlie the tenants of the CTM: with weight information provided daily, adjustments can be made to intake or expenditure to control body weight. The feedback provided by the weight chart is theorized to reinforce behaviors that cause weight to move in the intended direction, allowing participants to make changes in their eating or activity that best fit their lifestyle. This kind of flexible restraint has been found to be more closely related to successful dieting than the conventional type of dieting [28].

The rationale for using slow weight loss, guided by 1% decrements, with the CTM is that people initially try several techniques to reduce their energy intake or increase their energy expenditure. All work in the short term, but sustaining a weight loss requires that the person finds those behavioral changes they can live with over a prolonged, if not indefinite, period of time.

### 4.1. Limitations and Contributions

This study has a number of limitations. Participants were self-selected individuals interested in losing weight. These individuals were members of a campus wellness organization suggesting that these people may have had a heightened concern about their health. Due to racial and ethnic homogeneity, generalizations about how the CTM may influence weight in diverse populations cannot be made from this study. Similarly, conclusions about different age ranges or stages in life (e.g., premenopausal versus postmenopausal) cannot be made. Also, only a small number (*n* = 6) of participants in the intervention group reached the suggested goal of 10% weight loss in the first year of the study. Information about personal weight loss goals and if these exceeded or were less than the researcher suggested 10% goal would have been helpful in providing information for why this goal was not met. It is possible that the self-directed nature of the CTM program only works for a small percentage of people to produce a 10% weight loss, while smaller losses (e.g., 2-3%) are possible for a larger percentage of people. The most concerning limitation is that we are unable to separate the degree to which the CTM was the factor causing the weight change versus the fact that participants were cognizant of the study team's oversight. We tried to keep investigator involvement and participants' desire to please the investigator at a minimum; no rewards were provided or congratulatory remarks were sent as a rule when stage changes were made. Despite these efforts, for many participants, knowing that someone was watching them may have influenced study engagement and, therefore, weight loss over the course of the study.

Despite these limitations, this study makes a meaningful contribution to the existing work on weight control. This is the only weight loss study we are aware of that focuses its intervention uniquely on self-weighing (and visual feedback) without the addition of weight loss education lessons as comparable studies tend to use more of a comprehensive approach (e.g., [8]). Since the control group and experimental group received identical information about weight loss strategies at the initial session (with the exception of the description of the CTM) this factor can be ruled out as having contributed to the weight effects.

This study tested a low-cost and low-intensity intervention that can be disseminated easily through the internet. This type of program would be feasible for healthcare practitioners to carry out with a moderate number of patients, allowing them to allocate their time to those that require more support. From the patient's perspective, this would enable one to manage one's own weight while knowing that the process is being overseen. Most importantly, the small weight losses achieved with using the CTM were maintained during the second year of the study.

## 5. Conclusion

In a society that has seen body weights increasing for several decades, techniques to reduce weight, even minimally, and sustain this reduction are important. As little as 5% weight loss is clinically significant [29]. The intervention group in this study lost about half of this amount using the CTM intervention. Self-weighing and visual feedback may be a useful strategy combined with other techniques to promote healthful weight loss.

## Figures and Tables

**Table 1:** Baseline characteristics for total sample and by treatment group.

Baseline characteristics				
	Total	Control	CTM intervention	*p* value diff.^a^
Age (years)	46.6 ± 9.6^b^ (*n* = 144)^c^	48.2 ± 9.9 (*n* = 66)	45.3 ± 9.6 (*n* = 77)	0.071
BMI (kg/m^2^)	33.5 ± 5.0 (*n* = 148)	33.7 ± 5.1 (*n* = 68)	33.4 ± 5.1 (*n* = 81)	0.898
Body weight (kgs)	93.8 ± 17.4 (*n* = 148)	93.1 ± 17.9 (*n* = 68)	94.3 ± 17.0 (*n* = 81)	0.690
Body weight (lbs)	206.7 ± 38.3 (*n* = 149)	205.3 ± 39.5 (*n* = 68)	207.8 ± 37.4 (*n* = 81)	0.690
Height (in)	65.7 ± 3.7 (*n* = 142)	65.3 ± 3.7 (*n* = 65)	66.1 ± 3.7 (*n* = 77)	0.199
Female^d^ (*n*, %)	122, 81.9	59, 86.8	63, 77.8	0.156
Education (years), *highest level of education completed *	15.9 ± 2.2 (*n* = 146)	16.0 ± 2.2 (*n* = 67)	15.8 ± 2.2 (*n* = 79)	0.454
Ethnicity (number of participants)				
American Indian	3	2	1	e
Asian	2	2	0	
African American	6	3	3	
Hispanic	1	1	0	
White	144	65	79	0.696
Other	2	0	2	

^a^ *p* value for the difference between control and experimental group means (independent samples, 2-tailed test or chi-squared 2-tailed test).

^b^Mean ± standard deviation.

^c^ *n* may vary because of different data collection mechanisms (body weight taken in person, age reported via online survey).

^d^For questions with yes/no answers, the percentage that reported “yes” is shown; the *p* value column displays the *p* value of the chi-squared statistics for a two-tailed test.

^e^When the expected cell count is less than 5, the chi-squared statistics cannot be calculated.

**Table 2:** Comparison between present results and studies that have observed weight changes for one year following treatment.

Study	Observation (subgroup)	Percent weight regained
**Present study**	**CTM**	−**0.85**
Sherwood et al. [30]	Guided	5.09
Richelsen et al. [31]	Orlistat	5.09
Wing et al. [16]	Face to face	6.50
Perri et al. [32]	Face-to-face extended care	11.88
Perri et al. [32]	Telephone extended care	12.77
Cussler et al. [33]	Internet	13.21
Perri et al. [34]	Behavioral therapy + exercise + maintenance training	13.54
Wing et al. [16]	Control	16.13
Wing et al. [16]	Internet	16.15
Sherwood et al. [30]	Self-guided	16.93
Davidson et al. [35]	Orlistat-orlistat	17.83
Perri et al. [34]	Problem-solving	18.29
Cussler et al. [33]	Self-directed	19.23
Perri et al. [34]	Behavioral therapy + maintenance training	19.26
Sjöström et al. [36]	Orlistat-orlistat	19.65
Richelsen et al. [31]	Placebo	20.70
Harvey-Berino et al. [37]	In person	21.69
Davidson et al. [35]	Orlistat-low dose	24.58
Sjöström et al. [36]	Placebo-placebo	25.74
Sjöström et al. [36]	Placebo-orlistat	28.04
Perri et al. [34]	Relapse prevention training	28.16
Davidson et al. [35]	Placebo	33.53
Perri et al. [32]	Education	35.24
Harvey-Berino et al. [37]	Internet	36.23
Kramer et al. [38]	Control	36.79
Kramer et al. [38]	Skills focused	38.76
Sjöström et al. [36]	Orlistat-placebo	41.53
Harvey-Berino et al. [37]	Minimum personal	41.67
Stevens et al. [39]	All	45.45
Kramer et al. [38]	Weight focused	46.69
Stevens et al. [39]	White men	47.27
Stevens et al. [39]	White men and women	48.98
Stevens et al. [39]	All men	50.98
Davidson et al. [35]	Orlistat-placebo	51.42
Stevens et al. [39]	White women	52.78
Perri et al. [40]	Behavioral therapy + exercise	54.07
Perri et al. [34]	No further training	60.88
Stevens et al. [39]	All women	61.29
Perri et al. [40]	Behavioral therapy	76.19
Stevens et al. [39]	Black women	80.95
Stevens et al. [39]	All Blacks	91.30
Stevens et al. [39]	Black men	100.00
